# Dahonggou Creek virus, a divergent lineage of hantavirus harbored by the long-tailed mole (*Scaptonyx fusicaudus*)

**DOI:** 10.1186/s41182-016-0017-6

**Published:** 2016-06-20

**Authors:** Hae Ji Kang, Se Hun Gu, Joseph A. Cook, Richard Yanagihara

**Affiliations:** Pacific Center for Emerging Infectious Diseases Research, Departments of Pediatrics and Tropical Medicine, Medical Microbiology and Pharmacology, John A. Burns School of Medicine, University of Hawaii at Manoa, Honolulu, HI USA; Museum of Southwestern Biology, Department of Biology, University of New Mexico, Albuquerque, NM USA

**Keywords:** Hantavirus, Talpid, Evolution

## Abstract

Novel hantaviruses, recently detected in moles (order Eulipotyphla, family Talpidae) from Europe, Asia, and North America would predict a broader host range and wider ecological diversity. Employing RT-PCR, archival frozen tissues from the Chinese shrew mole (*Uropsilus soricipes*), broad-footed mole (*Scapanus latimanus*), coast mole (*Scapanus orarius*), Townsend’s mole (*Scapanus townsendii*), and long-tailed mole (*Scaptonyx fusicaudus*) were analyzed for hantavirus RNA. Following multiple attempts, a previously unrecognized hantavirus, designated Dahonggou Creek virus (DHCV), was detected in a long-tailed mole, captured in Shimian County, Sichuan Province, People’s Republic of China, in August 1989. Analyses of a 1058-nucleotide region of the RNA-dependent RNA polymerase-encoding L segment indicated that DHCV was genetically distinct from other rodent-, shrew-, mole-, and bat-borne hantaviruses. Phylogenetic trees, using maximum likelihood and Bayesian methods, showed that DHCV represented a divergent lineage comprising crocidurine and myosoricine shrew-borne hantaviruses. Although efforts to obtain the S- and M-genomic segments failed, the L-segment sequence analysis, reported here, expands the genetic database of non-rodent-borne hantaviruses. Also, by further mining natural history collections of archival specimens, the genetic diversity of hantaviruses will elucidate their evolutionary origins.

## Introduction

Hantaviruses are members of the family *Bunyaviridae*, all of whom possess a negative-sense, single-stranded tripartite RNA genome, consisting of large (L), medium (M), and small (S) segments, which encode an RNA-dependent RNA polymerase (RdRp), envelope glycoproteins (Gn and Gc), and a nucleocapsid (N) protein, respectively [1]. However, unlike other members of this large virus family which are carried by insects or arthropods, hantaviruses are hosted by small mammals, notably rodents (order Rodentia, families Muridae and Cricetidae), as well as shrews (order Eulipotyphla, family Soricidae), belonging to three subfamilies (Soricinae, Crocidurinae, and Myosoricinae), and moles (family Talpidae) of the Talpinae and Scalopinae subfamilies [2, 3]. Recently, insectivorous bats (order Chiroptera) have also been shown to serve as reservoirs of divergent lineages of hantaviruses [4–9]. To date, the pathogenic potential of the newfound shrew-, mole-, and bat-borne hantaviruses is unknown, whereas selected rodent-borne hantaviruses have been associated with acute-onset febrile diseases of varying clinical severity, known as hemorrhagic fever with renal syndrome and hantavirus pulmonary syndrome [10].

Among the newfound mole-borne hantaviruses are Asama virus (ASAV) in the Japanese shrew mole (*Urotrichus talpoides*) [11], Oxbow virus (OXBV) in the American shrew mole (*Neurotrichus gibbsii*) [12], and Rockport virus (RKPV) in the eastern mole (*Scalopus aquaticus*) [13]. Also, a divergent lineage of hantavirus, designated Nova virus (NVAV), has been identified in the widely distributed European mole (*Talpa europaea*) [14], in which very high prevalences of NVAV infection have been found in France [15] and Poland [16].

In testing archival frozen tissues from a natural history collection of moles, trapped in the People’s Republic of China and the USA, we now report the detection of a novel hantavirus, named Dahonggou Creek virus (DHCV), in the long-tailed mole (*Scaptonyx fusicaudus*).

## Methods

Tissues, stored frozen at −80 °C at the Museum of Southwestern Biology in Albuquerque, New Mexico, had been collected from five Chinese shrew moles (*Uropsilus soricipes*) and two long-tailed moles, captured in Shimian Xian, Sichuan Province, in the People’s Republic of China in 2005 and 1989, respectively, and from two broad-footed moles (*Scapanus latimanus*), two coast moles (*Scapanus orarius*), and two Townsend’s moles (*Scapanus townsendii*), in California (Sonoma) and Washington (Walla Walla and Gray’s Harbor) in 1984, according to well-established protocols, approved by the Institutional Animal Care and Use Committee of the University of New Mexico. Tissues were analyzed for hantavirus RNA by RT-PCR, using newly designed and previously employed oligonucleotide primers [12–16]. Briefly, total RNA was extracted from tissues using the PureLink Micro-to-Midi total RNA purification kit (Invitrogen, San Diego, CA), and cDNA was synthesized using the SuperScript III First-Strand Synthesis Systems (Invitrogen) with a highly conserved primer and/or random hexamers by two-step RT-PCR cycles. First- and second-round PCR were performed in 20-μL reaction mixtures, containing 250 μM dNTP, 2.5 mM MgCl_2_, 1 U of Takara LA Taq polymerase (Takara, Shiga, Japan), and 0.25 μM of each primer. Initial denaturation at 94 °C for 2 min was followed by two cycles each of denaturation at 94 °C for 30 s, two-degree step-down annealing from 46 to 38 °C for 40 s, and elongation at 72 °C for 1 min, then 30 cycles of denaturation at 94 °C for 30 s, annealing at 42 °C for 40 s, and elongation at 72 °C for 1 min, in a GeneAmp PCR 9700 thermal cycler (PerkinElmer, Waltham, MA). PCR products were separated, using MobiSpin S-400 spin columns (MoBiTec, Goettingen, Germany), and amplicons were sequenced directly using an ABI Prism 3130 Genetic Analyzer (Applied Biosystems, Foster City, CA).

Phylogenetic trees were generated using maximum likelihood and Bayesian methods, implemented in the RAxML Blackbox webserver [17] and MrBayes 3.1 [18], under the best-fit GTR+I+Γ model of evolution selected by hierarchical likelihood-ratio test in MrModeltest v2.3 [19] and jModelTest version 0.1 [20]. Two replicate Bayesian Metropolis-Hastings Markov chain Monte Carlo runs, each consisting of six chains of 10 million generations sampled every 100 generations with a burn-in of 25,000 (25 %), resulted in 150,000 trees overall. Topologies were evaluated by bootstrap analysis of 1000 iterations (implemented in RAxML Blackbox), and posterior node probabilities were based on 2 million generations and estimated sample sizes over 100 (implemented in MrBayes).

## Results and discussion

**Fig. 1:**
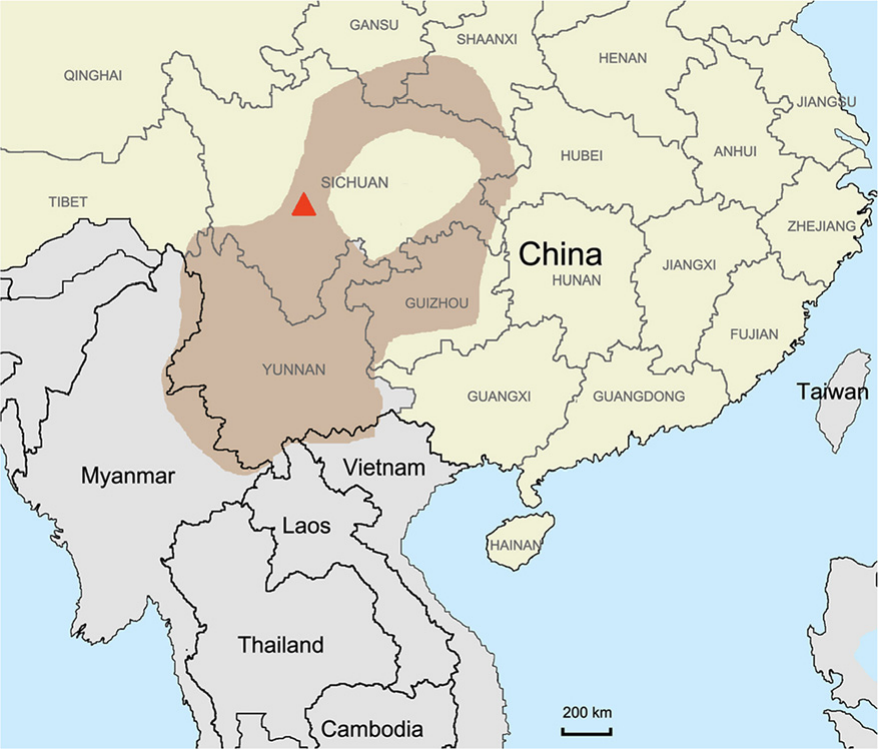
Map of the People’s Republic of China and neighboring countries, showing the site in Sichuan province, where the hantavirus-infected long-tailed mole (*Scaptonyx fusicaudus*) was captured (*red triangle*). The *shaded area* designates the geographic distribution of the long-tailed mole

By employing oligonucleotide primers and PCR cycling conditions used successfully to detect other mole-borne hantaviruses [12–16], a 1058-nucleotide region of the RdRp-encoding L segment was amplified from RNA extracted from heart and kidney tissues of a long-tailed mole, captured along Dahonggou Creek (29° 08′ N, 102° 25′ E), 17 km south-southeast of Shimian, in Sichuan Province, in China, on August 11, 1989 (Fig. 1). Countless attempts to amplify the S and M segments were unsuccessful. Apart from the inability to design suitable primers because of the vast genetic diversity, the limited amount of tissue and presumably poor quality of RNA proved to be insurmountable obstacles. Similarly, repeated RT-PCR attempts failed to detect hantavirus RNA in tissues from the other mole species. That said, based on our past experience of initially failing then succeeding to amplify hantavirus genes from other mole tissues [11–14], we are not entirely convinced that these other mole species do not harbor hantaviruses.

The whole genomes of many hantaviruses, previously described and recently discovered, are unavailable. A 1-kb sequence from a single specimen is far from optimal and full-length genomes from multiple long-tailed moles would be required to gain a more definitive conclusion about the molecular phylogeny and genetic diversity of DHCV. However, while admittedly incomplete, the sequence analysis presented in this report would facilitate and guide future studies by other investigators, seeking to expand the genetic database of non-rodent-borne hantaviruses. Also, completion of the DHCV genome from tissues of more recently collected long-tailed moles, using next-generation sequencing technology, is warranted.

Pairwise alignment and comparison of the partial L-segment sequence indicated that DHCV probably represents a distinct hantavirus species, with the highest sequence similarity of approximately 75 and 80 % at the nucleotide and amino acid levels, respectively, with Thottapalayam virus (TPMV) [21] and Imjin virus (MJNV) [22] in Asian crocidurine shrews and with Uluguru virus (ULUV) and Kilimanjaro virus (KMJV) in African myosoricine shrews [23] (Table 1). Direct comparison between DHCV strain MSB281632 (GenBank HQ616595) and hantavirus YN05-47 (GenBank JF915719), detected in a long-tailed mole from northwestern Yunnan province, was not possible, because they were of different regions of the L segment. However, phylogenetic analysis indicated that YN05-47 was more closely related to Jeju virus [24], a hantavirus harbored by the Asian lesser white-toothed shrew (*Crocidura shantungensis*), and may represent spillover from syntopic hantavirus-infected shrews. Alternatively, long-tailed moles might harbor more than one distinct hantavirus, as suggested by recent studies in Iberian moles (*Talpa occidentalis*) (Gu et al., unpublished observations).

**Table 1:** Nucleotide and amino acid sequence similarity (%) between DHCV strain MSB281632 and representative rodent-, shrew-, mole-, and bat-borne hantaviruses

		DHCV MSB281632	
Virus	Strain	1058 nt	352 aa
NVAV	MSB95703	69.4	71.9
OXBV	Ng1453	69.1	72.7
ASAV	N10	71.0	74.4
RKPV	MSB57412	71.4	75.8
MJNV	Cl 05-11	73.3	79.8
TPMV	VRC66412	72.4	78.7
KMJV	FMNH174124	75.2	80.1
ULUV	FMNH158302	74.3	79.3
MOYV	KB576	68.4	75.0
XSV	VN1982B4	70.5	74.1
CBNV	CBN-3	70.2	74.4
JMSV	MSB144475	69.2	72.4
KKMV	MSB148794	66.7	72.7
SWSV	mp70	67.8	73.0
ARTV	MSB148558	68.7	73.0
JJUV	10-11	66.5	70.2
AZGV	KBM15	69.5	72.2
HTNV	76-118	68.5	73.0
SOOV	SOO-1	67.5	71.9
SEOV	80-39	68.9	73.9
DOBV	Greece	68.2	75.0
SNV	NMH10	69.8	72.7
ANDV	Chile9717869	68.9	73.3
PUUV	Sotkamo	69.8	73.9
TULV	M5302v	71.1	72.2
PHV	PH-1	66.6	71.9

*Abbreviations*: *ANDV* Andes virus, *ARTV* Artybash virus, *ASAV* Asama virus, *AZGV* Azagny virus, *CBNV* Cao Bang virus, *DOBV* Dobrava virus, *HTNV* Hantaan virus, *JJUV* Jeju virus, *JMSV* Jemez Spring virus, *KKMV* Kenkeme virus, *KMJV* Kilimanjaro virus, *MJNV* Imjin virus, *MOYV* Mouyassué virus, *NVAV* Nova virus, *OXBV* Oxbow virus, *PHV* Prospect Hill virus, *PUUV* Puumala virus, *RKPV* Rockport virus, *SEOV* Seoul virus, *SNV* Sin Nombre virus, *SOOV* Soochong virus, *SWSV* Seewis virus, *TPMV* Thottapalayam virus, *TULV* Tula virus, *ULUV* Uluguru virus, *XSV* Xuan Son virus, *nt* nucleotides, *aa* amino acids

**Fig. 2:**
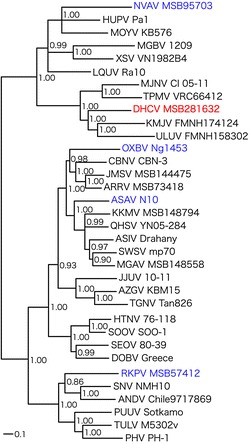
Phylogenetic tree was generated by the maximum likelihood and Bayesian methods, based on the alignment of the L-segment sequences of Dahonggou Creek virus (DHCV) strain MSB281632 (labeled in *red*) and other hantaviruses. Since tree topologies were very similar using RAxML and MrBayes, the tree generated by MrBayes was displayed. The phylogenetic position of DHCV (HQ616595) (labeled in *red*) is shown in relationship to other mole-borne hantaviruses (labeled in *blue*), including Asama virus (ASAV N10, EU929078), Oxbow virus (OXBV Ng1453, FJ593497), Nova virus (NVAV MSB95703, FJ593498), and Rockport virus (RKPV MSB57412, HM015221). Also shown are the phylogenetic positions of bat-borne hantaviruses, including Mouyassué virus (MOYV KB576, JQ287716), Xuan Son virus (XSV VN1982B4, JX912953), Huangpi virus (HUPV Pa-1, JX465369), Magboi virus (MGBV 1209, JN037851), Longquan virus (LQUV Ra-10, JX465379); shrew-borne hantaviruses, including Thottapalayam virus (TPMV VRC66412, EU001330), Imjin virus (MJNV Cl 05-11, EF641806), Uluguru virus (ULUV FMNH158302, JX193697), Kilimanjaro virus (KMJV FMNH174124, JX193700), Seewis virus (SWSV mp70, EF636026), Cao Bang virus (CBNV CBN-3, EF543525), Jemez Springs virus (JMSV MSB144475, FJ593501), Kenkeme virus (KKMV MSB148794, GQ306150), Amga virus (MGAV MSB148558, KM201413), Ash River virus (ARRV MSB73418, EF619961), Asikkala virus (ASIV Drahany, KC880348), Qian Hu Shan virus (QHSV YN05-284, GU566021), Tanganya virus (TGNV Tan826, EF050454), Azagny virus (AZGV KBM15, JF276228), Jeju virus (JJUV 10-11, HQ834697); and rodent-borne hantaviruses, including Hantaan virus (HTNV 76-118, NC_005222), Soochong virus (SOOV SOO-1, DQ056292), Dobrava virus (DOBV Greece, NC_005235), Seoul virus (SEOV 80-39, NC_005238), Tula virus (TULV M5302v, NC_005226), Puumala virus (PUUV Sotkamo, NC_005225), Prospect Hill virus (PHV PH-1, EF646763), Sin Nombre virus (SNV NMH10, NC_005217), and Andes virus (ANDV Chile9717869, AF291704). The numbers at each node are posterior node probabilities based on 150,000 trees. The *scale bar* indicates nucleotide substitutions per site

Phylogenetic analyses indicated four distinct clades, with DHCV positioned in a divergent lineage comprising TPMV, MJNV, ULUV, and KMJV (Fig. 2). As evidenced by their phylogenetic positions in each of the four hantavirus clades, mole-borne hantaviruses may be somewhat more catholic in their host selection than present-day rodent-borne hantaviruses [2]. Whether or not this signifies that ancestral moles served as the early hosts of primordial hantaviruses requires further investigation.

The fossorial long-tailed mole, which closely resembles the American shrew mole and Japanese shrew mole in size and appearance, as well as ecological habits [25], is restricted to high altitudes (2000–4100 m) in montane coniferous forests in central and southern China, extending to northern Myanmar and northern Vietnam. It represents the only species within the *Scaptonyx* genus. Because the long-tailed mole is sympatric with *Uropsilus* moles, the latter might also serve as a potential reservoir host of hantaviruses. Thus, efforts to test tissues from additional *Uropsilus* moles are also warranted.

Museum curators and field mammalogists, who willingly granted access to their priceless archival tissue collections, have contributed greatly to the acquisition of new knowledge about the geospatial distribution and temporal dynamics, host range, and genetic diversity of hantaviruses in shrews, moles, and insectivorous bats [2, 3]. The availability of these specimens, which were not originally intended for our studies, provide compelling justification for the continued expansion and long-term maintenance of natural history tissue repositories for future investigations, including virus-discovery efforts to better catalog the vast environmental microbiome. In so doing, important improvements could be made in preparedness and response to new and emerging zoonotic infectious diseases [3].
